# Combination Therapy With Histone Deacetylase Inhibitors (HDACi) for the Treatment of Cancer: Achieving the Full Therapeutic Potential of HDACi

**DOI:** 10.3389/fonc.2018.00092

**Published:** 2018-03-29

**Authors:** Amila Suraweera, Kenneth J. O’Byrne, Derek J. Richard

**Affiliations:** ^1^School of Biomedical Research, Institute of Health and Biomedical Innovation at the Translational Research Institute, Queensland University of Technology, Brisbane, QLD, Australia; ^2^Princess Alexandra Hospital, Brisbane, QLD, Australia

**Keywords:** cancer, chemotherapeutic drugs, histone deacetylases, histone deacetylase inhibitors, combination therapy

## Abstract

Genetic and epigenetic changes in DNA are involved in cancer development and tumor progression. Histone deacetylases (HDACs) are key regulators of gene expression that act as transcriptional repressors by removing acetyl groups from histones. HDACs are dysregulated in many cancers, making them a therapeutic target for the treatment of cancer. Histone deacetylase inhibitors (HDACi), a novel class of small-molecular therapeutics, are now approved by the Food and Drug Administration as anticancer agents. While they have shown great promise, resistance to HDACi is often observed and furthermore, HDACi have shown limited success in treating solid tumors. The combination of HDACi with standard chemotherapeutic drugs has demonstrated promising anticancer effects in both preclinical and clinical studies. In this review, we summarize the research thus far on HDACi in combination therapy, with other anticancer agents and their translation into preclinical and clinical studies. We additionally highlight the side effects associated with HDACi in cancer therapy and discuss potential biomarkers to either select or predict a patient’s response to these agents, in order to limit the off-target toxicity associated with HDACi.

## Introduction

Genetic and genomic alterations as well as epigenetic modifications of DNA are involved in cancer development and tumor progression. Epigenetic changes modify chromatin structure and the accessibility of DNA, thus regulating patterns of gene expression. Epigenetic mechanisms include covalent histone modifications, DNA methylation, non-covalent mechanisms including the incorporation of histone variants and nucleosome remodeling, and non-coding RNAs. The N-terminal tail of histones can be modified posttranslationally by acetylation, methylation, ubiquitination, phosphorylation, sumoylation, ADP ribosylation, deamination, and proline isomerization (1–3).

Anticancer agents currently used in the clinic, including cytotoxic chemotherapy, targeted therapies, and immunotherapy have played a tremendous role in improving patient survival, symptom control, and quality of life. However, most cytotoxic drugs have a narrow therapeutic index. Cancers eventually develop drug resistance to the majority of systemic therapies. Furthermore, anticancer agents target normal cells in addition to the cancer cells, resulting in toxicities either due to direct cell damage or, in the case of immunotherapy, to inappropriate activation of autoimmune illnesses. Indeed, as cytotoxic drugs target naturally regenerating tissues, mainly the bone marrow and gastrointestinal tract, the formation of secondary hematologic and solid tumors may be seen many years following treatment (4–7). Combining anticancer drugs with other chemotherapeutic agents is often used to maximize efficacy, while reducing toxicity and resistance by administering lower drug doses, and their combination has shown synergistic or additive antitumor effects (8–11). This review focuses on research thus far on histone deacetylase inhibitors (HDACi) in combination therapy with other chemotherapeutic agents and how this understanding can be utilized to optimize their application as anticancer agents in the clinic.

## Histone Deacetylases (HDACs) and HDACi

**Figure 1:**
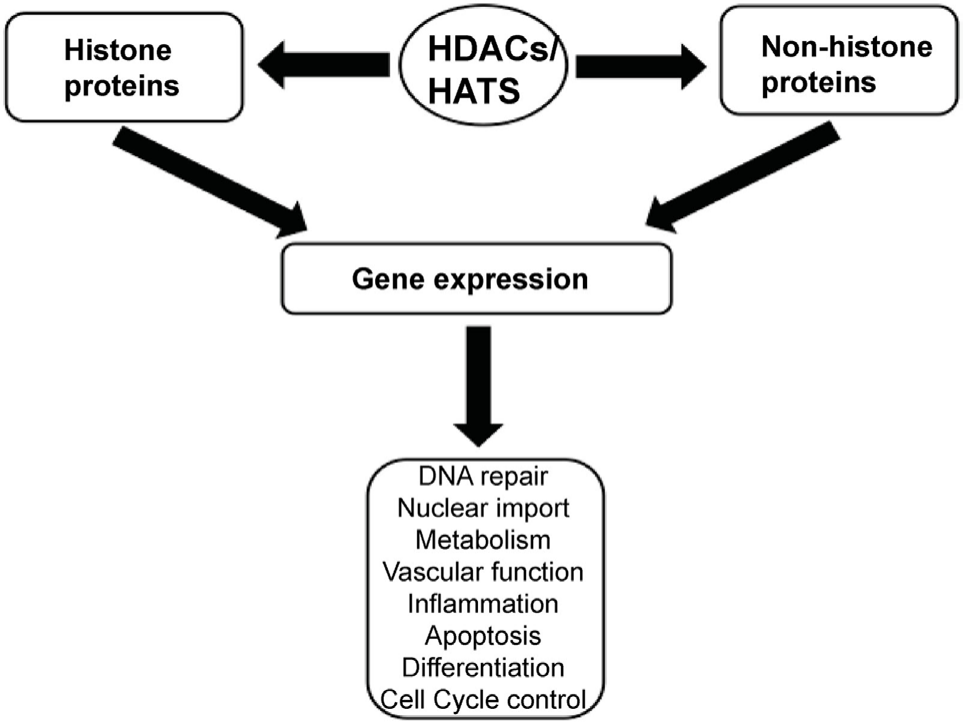
A schematic illustration depicting the central role of histone deacetylases (HDACs) and histone acetyltransferases (HATs) in regulating gene expression.

Histone acetylation is one the most extensively studied posttranslational covalent modifications of histones that is regulated by the opposing actions of histone acetyltransferases (HATs) and HDACs. While HATs mediate the acetylation of lysine residues associated with gene transcription, HDACs remove the acetyl group from the positively charged histone lysine residues, enabling the negatively charged DNA to bind to the nucleosome proteins. HDACs are critical regulators of gene expression that act as transcriptional repressors while histone acetylation results in a more relaxed chromatin confirmation, enabling transcriptional activation. This opposing action of HATs and HDACs enable the regulation of gene expression in response to the environment (Figure 1) (12–15). To date, in humans there are 18 HDACs that have been identified which are classified according to their homology to yeast HDACs. The HDAC Classes I, II, and IV are Zn^2+^-dependent metalloproteins, while Class III HDACs are NAD^+^-dependent (15, 16). Class I HDACs are related to yeast *RPD3* gene and include HDAC1, 2, 3, and 8. On the other hand, HDACs 4, 5, 7, and 9 are classified as Class IIa HDACs, while HDACs 6 and 10 belong to Class IIb and are related to the yeast *Hda1* gene (17). Class III (sirtuins) include SIRT1–7 and are related the *Sir2* gene while Class IV contains HDAC11 (18, 19).

**Figure 2:**
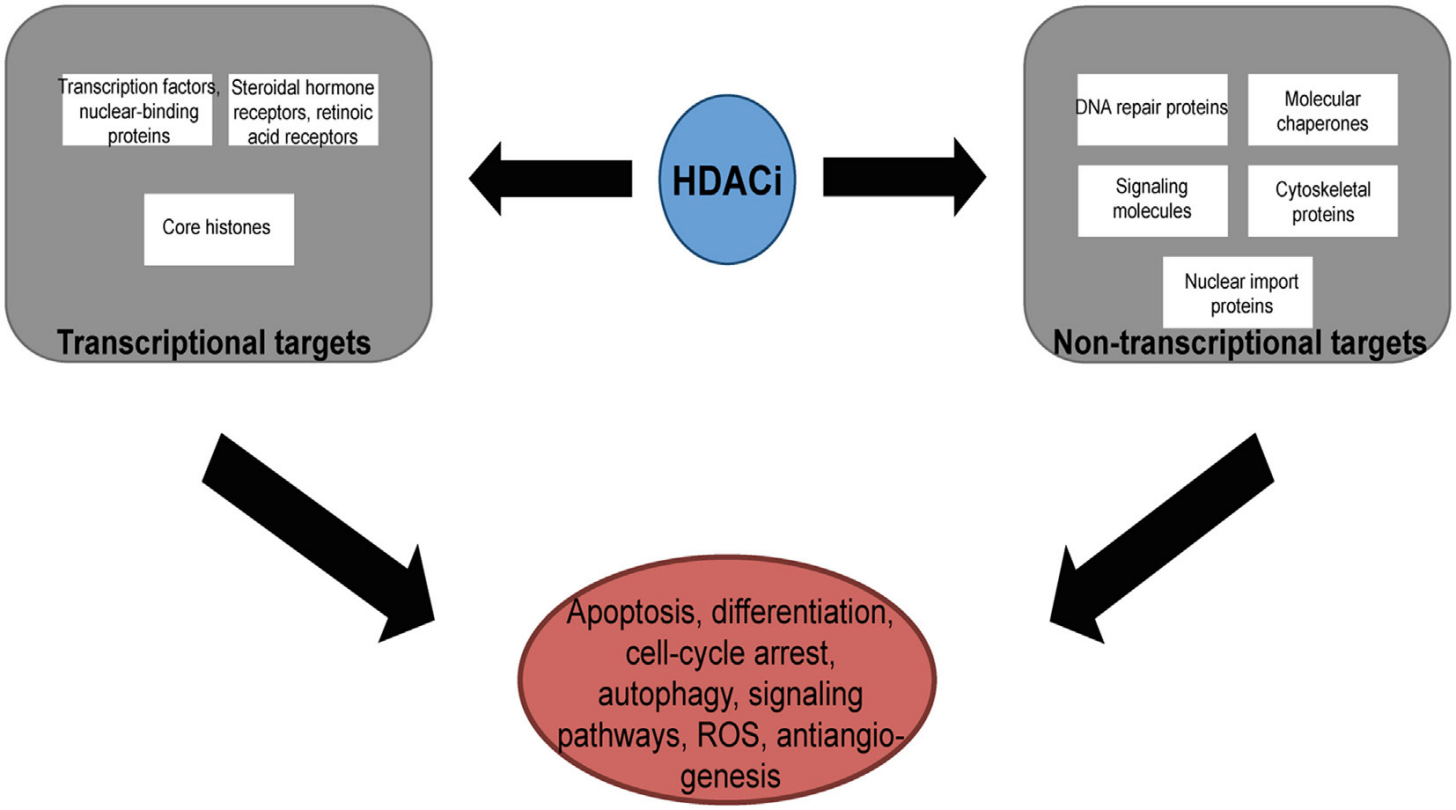
The pleotropic cellular effects of histone deacetylase inhibitors (HDACi). The modification of the acetylation status of cores histones and non-histone proteins result in the multiple cellular effects seen with HDACi. ROS, reactive-oxygen species.

Histone deacetylases are often dysregulated in numerous disorders, including cancer, thereby influencing gene transcription and affecting normal cellular behavior (13, 20). High expression levels of HDAC1, 2, and 3 have been shown to be associated with poor patient outcomes in gastric and ovarian cancers (21, 22), while high expression of HDAC8 is correlated with poor survival and advanced disease in neuroblastoma (23). Additionally, dysregulation of HDAC1 expression was demonstrated to correlate with poor prognosis in multiple myeloma (24). HDACi are currently used in the clinic as anticancer agents and are a powerful new class of small-molecular therapeutics that alters the regulation of histone and non-histone proteins. HDACi increase the acetylation of core histones, leading to an open chromatin confirmation that is more accessible to DNA-targeting agents. HDACi have pleiotropic cellular effects (Figure 2) and induce the expression of pro-apoptotic genes/proteins, cause cellular differentiation and/or cell cycle arrest (13, 15, 19, 25, 26). The classes of HDACs and the HDACi that are targeted against them are summarized in Table 1.

**Table 1:** Classification of histone deacetylases (HDACs) and the histone deacetylase inhibitors (HDACi) that target them.

Class	Members	Cellular function	Histone deacetylase inhibitor
I	HDAC1	Cell survival and proliferation	Vorinostat, panobinostat, belinostat, ITF2357, PCI-24781, FK228, entinostat, MGCD0103, phenyl butyrate, valproic acid, trichostatin A, LAQ824, mocetinostat, pracinostat
I	HDAC2	Cell proliferation and insulin resistance	
I	HDAC3	Cell survival and proliferation	
I	HDAC8	Cell proliferation	

IIA	HDAC4	Regulation of skeletogenesis and gluconeogenesis	Vorinostat, pracinostat, panobinostat, belinostat, ITF2357, PCI-24781, phenyl butyrate, valproic acid, trichostatin A, LAQ824
IIA	HDAC5	Cellular development and differentiation, cardiovascular growth and function, gluconeogenesis	
IIA	HDAC7	Thymocyte differentiation, endothelial function and glucogenesis	
IIA	HDAC9	HR, thymocyte differentiation, cardiovascular growth and function	

IIB	HDAC6	Cell motility and control of cytoskeletal dynamics	Vorinostat, panobinostat, belinostat, IFT2357, PCI-24781, trichostatin A, LAQ824, pracinostat
IIB	HDAC10	HR, autophagy mediated cell survival	

III	SIRT1	Apoptosis, aging, redox regulation, and autoimmune system regulation	Nicotinamide (all of Class III)Cambinol, tenovin 1, tenovin 6, sirtinol, EX-527 (SIRT1 and 2 only)
III	SIRT2	Mitotic exit of cell cycle, regulation of mitotic checkpoint under stress and neuronal motility and differentiation	
III	SIRT3	Regulation of energy metabolism, apoptosis and cell signaling	
III	SIRT4	Regulation of insulin secretion, ATP regulation, metabolism, apoptosis, and cell signaling	
III	SIRT5	Regulation of urea cycle, energy metabolism, and ATP regulation	
III	SIRT6	Metabolic regulation	
III	SIRT7	Apoptosis	

IV	HDAC11	Immunomodulators-DNA replication	Vorinostat, trichostatin, LAQ824, belinostat, IFT2357, mocetinostat, pracinostat

## HDACi That have been Approved by the USA FDA

In 2006, SAHA (Vorinostat, Zolinza™, Merck & Co, Inc., USA) became the first HDACi to be approved by the FDA for the treatment of cutaneous T-cell lymphoma (CTCL) (27). FK228 (Romidepsin, Istodax™, Celgene Corp., USA), a cyclic peptide was later approved in 2009 for treating CTCL in patients who had received at least one prior systemic therapy (28). More recently, belinostat (PXD101, BELEODAQ™, Spectrum Pharmaceuticals, Inc.) was approved by the FDA in 2014 for the use against peripheral T-cell lymphoma (PTCL) (29) and panobinostat (Farydak, Novartis Pharmaceuticals) was licensed in 2015 for the treatment of multiple myeloma (30). A summary of HDACi that are currently approved by the FDA and/or are in clinical trials for the treatment of cancer are shown in Table 2.

**Table 2:** Histone deacetylase inhibitors (HDACi) that are currently approved by the FDA or in clinical trials for the treatment of cancer.

HDACi	Approved by the FDA	Approved elsewhere	In phase I/II/III clinical trials	Type of cancer targeted against
Vorinostat (SAHA)	Yes			Cutaneous T-cell lymphoma (CTCL) (2006)
Romidepsin (FK288)	Yes			CTCL (2009)
Chidamide		China		Peripheral T-cell lymphoma (PTCL)
Panobinostat (LBH589)	Yes		III	Multiple myeloma (FDA approved 2015) and CTCL
Belinostat (PXD101)	Yes			PTCL (2014)
Valproic acid			III	Cervical and ovarian
Tacedinaline (CI994)			III	Multiple myeloma and lung cancer
Mocetinostat			II	Follicular and Hodgkin lymphoma and acute myeloid leukemia
Abexinostat (PCI24781)			II	Sarcoma and lymphoma
(MS275-SNDX-275)			II	Hodgkin lymphoma, lung and breast cancer
Practinostat (SB939)			II	Recurrent or metastatic prostate cancer
Resminostat (4SC201)			II	Hodgkin lymphoma and hepatocellular carcinoma
Givinostat (IFT2357)			II	Refractory leukemia and myeloma
Quisinostat (JNJ-26481585)			I/II	Multiple myeloma, solid tumors
HBI-8000		China		PTCL
Kevetrin			I	Ovarian cancer and spleen metastasis
CUDC-101			I	Head and neck squamous carcinoma
AR42			I	Multiple myeloma, chronic lymphocytic leukemia or lymphoma
Tefinostat (CHR-2845)			I	Hematological malignancies
CHR-3996			I	Refractory solid tumors
4SC202			I	Colorectal cancer
CG200745			I	Solid tumors
Rocilinostat (ACY1215)			I	Multiple myeloma
ME-344			I	Solid refractory tumors

## HDACi Resistance and Rational for Combination Therapy

Resistance to HDACi is often seen and the basis of this resistance remains largely unknown. High levels of Bcl-2, Trx, and peroxiredoxins have previously been implicated with resistance of transformed cells to chemotherapy and may form the basis of resistance seen with HDACi (31–33). Increased expression of the cell cycle protein, p21, elevated levels of thioredoxin leading to lower reactive-oxygen species (ROS)-mediated DNA damage, constitutive activation of NFκB through p65 acetylation and elevated expression of the signaling proteins, MAPK, PI3K, or STAT3, have additionally been suggested to contribute toward the resistance of HDACi (34). Furthermore, chromatin and epigenetic alterations, changes in the expression levels of HDACs and changes in drug efflux mechanisms have been implicated as factors in the resistance to HDACi (35). It was recently speculated that the resistance to HDACi is a critical evolutionary consequence of environmental exposure to HDACi and that cancers that respond to HDACi have developed mutations that alters how the cancer cell responds to the HDACi. Hence, the identification of these mutations may enable the selective targeting of individual cancers susceptible to HDACi (36). Despite the approval by the FDA for the treatment of certain cancers, HDACi have been shown to have a limited therapeutic efficacy against solid tumors as a single therapeutic agent. HDACi have, however, been shown to function synergistically with a range of structurally and functionally diverse chemical compounds, biologically active polypeptides, and novel immune therapies. Combining HDACi with other cancer therapeutics may thus be an avenue to achieve their full therapeutic potential (19, 37, 38).

## HDACi in Combination with Drugs That Target DNA Repair Pathways

DNA double-strand breaks (DSBs) are among the most harmful lesions to cells, as the incorrect repair of just one DSB can lead to chromosomal fragmentation and rearrangements. Upon the induction of DSBs, the DNA damage response plays a crucial role in maintaining genomic integrity. This prevents the development of cancer through cell cycle checkpoint arrest and ensures that the DNA repair occurs prior to the resumption of the cell cycle. Cells have evolved two major pathways to repair DSBs, non-homologous-end joining (NHEJ), and homologous recombination. While NHEJ is a mechanism for rejoining broken DNA ends without the use of extensive homology and thus often regarded as being error-prone, by contrast, HR involves the copying of DNA from a homologous template. The repair mechanisms also act on different stages of the cell cycle. NHEJ is used at every stage of the cell cycle, while cells only use HR during the S and G2 phases of the cell cycle, when a sister chromatid is available. In HR, the protein kinase ataxia-telangiectasia mutated (ATM) is the key regulator of the cellular response to DSBs. Following the induction of DSBs, ATM is activated in a process that involves its auto-phosphorylation by the MRN (MRE11, Rad50, and NBS1) complex. ATM subsequently phosphorylates BRCA1, CHK2, and p53, resulting in the activation of response genes involved with multiple signaling pathways leading to the activation of cell cycle checkpoints, DNA repair, and apoptosis. Upon the induction of DSBs in NHEJ, the DNA-dependent protein kinase catalytic subunit (DNA-PKcs) and the Ku70/Ku80 heterodimer initially bind to the two ends of the DSB. These ends are subsequently modified by Artemis, ligated by ligase IV, and finally stabilized by XRCC4 and XLF (39–42).

The upregulation of the DNA damage response has been suggested to contribute toward the resistance of cancer cells to genotoxic therapies and thus, mitigating the DNA damage response represents an avenue to treat cancer cells more effectively. Indeed the selectivity of HDACi in specifically targeting cancer cells can be attributed to HDACi causing DNA damage that normal but not cancer cells can repair (43, 44). For example, the HDACi vorinostat induces DSBs in normal and LNCaP and A549 cancer cells (43). While γH2AX levels, a marker for the appearance of DSBs (45), increased in the cancer cells over time, in continued culture with vorinostat, they were found to decrease in the control, non-cancerous cells. Additionally, the authors showed that vorinostat suppressed the DNA repair proteins, MRE11 and RAD50, in only the cancer cells, collectively leading to cancer cell death.

Histone deacetylases have been recently shown to play a substantial role in attenuating the activation of ATM following DNA damage (46). Ataxia-telangiectasia patients, with defective ATM protein, display increased genomic instability, chromatin decondensation, and radiosensitivity (47). Interestingly, cells treated with HDACi display a similar phenotype (48). There is increasing evidence to suggest that HDACs regulate ATM. For instance, ATM has been shown to interact both *in vitro* and *in vivo* with HDAC1 and this interaction increases post-irradiation (46, 49). HDAC2 has been additionally shown to regulate the expression of chromatin remodeling proteins, including SMC1, an ATM substrate (50). More recently, research has shown that HDACs regulate ATM-mediated DNA damage signaling and established that HDAC1 and HDAC2 function as part of the DDR (46). The authors demonstrated that HDACi resulted in a reduced activation of ATM and its expression and decreased p53 activation both *in vitro* and *in vivo*. Thus, addition of HDACi followed by a subsequent treatment with DNA damaging agents led to an insufficient induction of the DDR, sustained DNA damage, and an increase in cell death. Olaparib (AZD-2281, Lynparza) is an oral PARP inhibitor, inhibiting the DNA repair protein poly ADP ribose polymerase. Olaparib is approved for the treatment of germline BRCA-mutated advanced ovarian cancer in patients who have received prior chemotherapy (51, 52). A recently reported phase III clinical trial has demonstrated that olaparib monotherapy is significantly superior to physicians choice of chemotherapy in the treatment of metastatic breast cancer (MBC) patients with germline BRAC1/2 mutations in terms of progression-free survival, time to second progression, objective response rate, and global health-related quality of life (ClinicalTrial.gov identifier: NCT02000622) (53). Additionally, there is a phase I/II clinical trial underway with the combination of olaparib and vorinostat. The aim of this study is to determine the safety and effectiveness of the combination of olaparib with high-dose chemotherapy (vorinostat, gemcitabine, busulfan, and melphalan, either with or without rituximab) in patients with refractory lymphomas (ClinicalTrial.gov identifier: NCT03259503).

In addition to their histone substrates, HDACi act on non-histone proteins including p53, Ku70, HSP90, NFκB, and α-tubulin (54). Thus, the acetylation of both histone and non-histone proteins give rise to the pleiotropic antitumor effects seen with HDACi at both epigenetic and cellular levels. By targeting the acetylation of Ku70, HDACi have additionally been shown to sensitize prostate cancer cells to DNA damaging agents. In this study, the authors demonstrated that the prostate cancer cells pretreated with the HDACi, trichostatin A, suberoylanilide hyroxamic acid, MS-275, and OSU-HDAC42, resulted in an increase in Ku70 acetylation which was followed by a lower DNA-binding affinity without the disruption of the Ku70/80 complex. When the prostate cancer cells were treated with the HDACi and the DNA damaging agents, bleomycin, doxorubicin, and etoposide, sequentially, the sensitization of the prostate cancer cells was more pronounced (55). Taken together, the combination of HDACi for sensitizing cancer cells with therapies that induce DNA damage warrants further clinical investigation.

## Combination of HDACi and Radiotherapy

To this day, radiotherapy continues to be among the most widely used cancer treatments, causing cell death by the induction of DSBs. HDACi enhance the radiosensitivity of cancer cells (56, 57). HDACi have been shown *in vitro* to lower a cell’s ability to repair IR-induced DNA damage, by affecting DNA damage signaling and the NHEJ and HR DSB repair pathways (17). In preclinical studies, HDACi have shown radiosensitizing effects with glioblastoma multiforme, melanoma, and head and neck squamous, colorectal, non-small cell lung, prostate, and MBCs (58). The first group to publish a case report on use of an HDACi with radiotherapy used valproic acid in combination with cisplatin and doxorubicin, 40 Gy radiation, and surgery to successfully treat a patient with anaplastic thyroid carcinoma (59). The novel pan-HDACi, panobinostat (LBH589), is currently being used in phase I and II clinical studies against various hematologic malignancies and solid tumors (60, 61). In combination with radiotherapy, LBH589 was shown to be an effective regimen for the treatment of prostate cancer *in vitro*. The authors demonstrated that LBH589 at low concentrations (IC_20_) in combination with radiation induced more apoptosis, resulted in a steady increase of the sub-G1 population of cells and led to the elimination of radiation-induced cell cycle arrest. Additionally, the combination treatment gave rise to more DNA damage and resulted in less activation of NHEJ and HR repair pathways (62). A phase I clinical trial (ClinicalTrial.gov identifier: NCT00455351) studying the treatment of vorinostat with short-term palliative pelvic radiotherapy showed that both treatments were tolerated in gastrointestinal tract carcinoma patients. Additionally, the combined treatment resulted in seven grade 3 adverse events in 16 of the patients receiving the combination. Further research is required to evaluate the efficacy and safety of the combination therapy. There are several completed and ongoing trials combining radiotherapy and various HDACi. These include a phase II trial to determine the efficacy of valproic acid with temozolomide and external beam radiation to treat high-grade gliomas (ClinicalTrial.gov identifier: NCT00302159), a phase I study of vorinostat in combination with palliative radiotherapy for patients with NSCLC (ClinicalTrial.gov identifier: NCT00821951), a phase I study of panobinostat combined with radiation therapy for treating prostate, esophageal, and head and neck cancers (ClinicalTrial.gov identifier: NCT00670553), and capeceitabine, vorinostat, and radiotherapy for the treatment of patients with non-metastatic pancreatic cancer (ClinicalTrial.gov identifier: NCT00983268).

## Dual Combination of HDACi and Topoisomerase Inhibitors

Topoisomerases are ubiquitous enzymes that play a vital role in replication, transcription, recombination, DNA repair, and chromatin remodeling, by over-winding or under-winding the DNA helix. There are two groups of topoisomerases, type I and II, which are classified according to their structure and mechanism of action. While type I topoisomerases cleave one strand of the DNA double helix reversibly, type II enzymes mediate strand passage through a double-strand DNA gate (63). Compared to monotherapy, combination therapies with HDACi and topoisomerase II inhibitors have lead to higher nuclear topoisomerase II inhibitor accumulation, an increase in DNA damage, growth inhibition, and cell death (64–67). Preclinical studies have shown greater efficacy when cells were pretreated with HDACi prior to exposure to DNA damaging agents (64, 68), similar to the decondensation of chromatin seen in cells from breast and other cancers (50, 69). The first human phase I dose-escalation clinical study combining valproic acid and the topoisomerase II inhibitor, epirubicin, in solid malignancies has recently been performed (70). This study showed that valproic acid is well tolerated and can be used in a clinical setting. The combination of valproic acid followed by epirubicin, resulted in an objective response rate of 22% and, additionally, 39% of patients had stable disease. Most significantly, this study showed responses in patients with anthracycline-resistant tumors and in heavily pretreated patients (ClinicalTrial.gov identifier: NCT00246103). In a pharmacokinetic and pharmacodynamic phase I/II study of valproic acid in combination with epirubicin/FEC, 64% of breast cancer patients had an objective response in the dose expansion cohort. This study demonstrated that the combination of valproic acid and epirubicin/FEC is safe, tolerable, and feasible (ClinicalTrial.gov identifier: NCT01010854) (71). The authors subsequently carried out a phase I combination trial of vorinostat and doxorubicin in treating patients with metastatic or advanced solid tumors (72). While a modest clinical effect was observed, this study showed that HDAC2 expression is a potential predictive biomarker and a target for developing isotype-specific inhibitors that may lead to greater inhibition with reduced toxicity (ClinicalTrial.gov identifier: NCT00331955). There are several completed and ongoing clinical trials involving HDACi and doxorubicin. A phase I/II dose-escalation trial of belinostat together with doxorubicin in patients with soft tissue sacrcoma (ClinicalTrial.gov identifier: NCT00878800) has just been completed. A dose-finding and dose-escalating phase I/II trial of vorinostat and pegylated liposomal doxorubicin in patients with advanced or refractory lymphoma (ClinicalTrial.gov identifier: NCT00785798) and a phase I trial studying the maximum tolerated dose of vorinostat in combination with bortezomib and doxorubicin hydrochloride liposome for the treatment of relapsed or refractory multiple myeloma (ClinicalTrial.gov identifier: NCT00744354) has just been terminated.

## HDACi in Combination with Platinum-Based Chemotherapeutics

*Cis*-diamminedichloroplatinum(II) (cisplatin) was first approved in 1978 for the treatment of bladder and testicular cancer (73, 74). Cisplatin has since been used as a first-line therapy for many different solid malignancies, including head and neck, ovarian, bladder, testicular, colorectal, bladder, cervical, and lung cancers, as a monotherapy or in combination with chemotherapeutic drugs. Cisplatin exerts its anticancer effects primarily through the generation of DNA lesions, followed by the induction of the DDR and mitochondrial apoptosis. Although cisplatin treatment results in many cases as an initial therapeutic success, chemoresistance eventually develops particularly in non-germ cell solid tumors (25, 75, 76). Thus, combining cisplatin with HDACi is a promising strategy to increase the efficacy of cisplatin (25). Concurrent SAHA (vorinostat) therapy has been shown to enhance tumor cell sensitivity to subtoxic doses of cisplatin in oral squamous cell carcinoma cell lines (77). The authors showed that the combination of both drugs synergistically induced cytotoxicity and apoptosis in the oral squamous cell carcinoma cells, compared to SAHA or cisplatin treatment alone. Trichostatin A was shown to synergistically enhance the antitumor response of cisplatin and additionally resensitize bladder cancer cells that were resistant to cisplatin (78) and valproic acid was shown to induce apoptosis and p16INK4A upregulation, thereby sensitizing melanoma cells to cisplatin and etoposide treatment (79).

Carboplatin (cyclobutane-1,1-dicarboxylate-*O*,*O*′) was developed as an analog of cisplatin that results in less nephron and neurotoxicity than cisplatin, but results in the same DNA adducts (80). A phase I clinical study was carried out to determine the safety and pharmacokinetics of the HDACi, belinostat, with carboplatin and/or paclitaxel in solid tumors. Of the 23 patients with solid tumors who were recruited in the study, six patients showed stable disease lasting ≥6 months, two patients had partial responses and one patient had a complete CA-125 response. Overall, belinostat with carboplatin and paclitaxel was well tolerated with no evidence of a pharmacokinetic interaction (81). A phase I pharmacokinetic study was additionally carried out with vorinostat in combination with carboplatin and paclitaxel for advanced solid malignancies (82, 83). This study demonstrated that this combination was tolerated well and of the 19 patients with NSCLC, 10 had partial responses and 4 patients showed stable disease. This study subsequently led to a phase II clinical study involving 94 patients with NSCLC with carboplatin and paclitaxel combined with either a placebo or vorinostat (ClinicalTrial.gov identifier: NCT01413750). The response rate, progression-free survival, and survival overall were significantly higher in those treated with vorinostat-carboplatin-paclitaxel compared to the group who received placebo-carboplatin-paclitaxel. The 1-year survival rate for the vorinostat-carboplatin-paclitaxel group compared to the placebo-carboplatin-paclitaxel group was 51% compared to 33%. Although vorinostat was found to improve the efficacy of carboplatin and paclitaxel, it also led to increased toxicity. Overall, this study demonstrated that vorinostat enhanced the efficacy of carboplatin and paclitaxel in patients with NSCLC patients (84, 85); however, the phase III trial with this combination was terminated due to lack of efficacy (ClinicalTrial.gov identifier: NCT00473889). A phase I trial with high-dose or low-dose vorinostat with carboplatin or paclitaxel for the treatment of advanced solid tumors is currently ongoing (ClinicalTrial.gov identifier: NCT01281176).

## Combining HDACi and Proteasome Inhibitors

The tightly regulated degradation of ubiquitinated proteins by the proteasome is essential in cells and impairment of the ubiquitin-proteasome system (UPS) leads to several pathological diseases (86). The UPS plays a key role in the cellular protein-degradation machinery and in preventing the accumulation of misfolded proteins (86–88). Cancer cells are dependent on the UPS as they are highly proliferative and have an increased requirement for protein synthesis, hence making them more vulnerable to proteasome inhibitors (89, 90). In 2003, the FDA approved bortezomib (PS-341, Velcade™, Millennium Pharmaceuticals Inc.) to treat relapsed and refractory multiple myeloma (91). However, peripheral neuropathy is often seen and is the most frequently associated dose limiting toxicity. The HDACi, vorinostat, romidepsin, dacinostat, and panobinostat, inhibit proliferation and lead to apoptosis of multiple myeloma cells and murine xenograft models. However, in phase I and phase II clinical trials, is has been observed that as a single agent, the activity of HDACi have been limited (92, 93). In several preclinical studies, synergistic antitumor effects were seen in multiple myeloma, when HDACi were combined with proteasome inhibitors, thus providing the rational for combining HDACi with these agents in clinical trials (94–97). The synergistic effect seen with combination of HDACi and proteasome inhibitors may be attributed to the multiple pathways targeting multiple myeloma cell biology. One prominent mechanism may be by disrupting protein degradation by inhibiting both the proteasome and aggresome with HDAC6i, resulting in the accumulation of significantly more polyubiquitinated proteins and causing increased cellular stress and apoptosis (94, 98, 99). In addition to multiple myeloma, investigators have demonstrated that combination therapy with bortezomib and the Class I HDACi, MS275, apicidin, and romidepsin, resulted in apoptosis *in vitro* and *in vivo* of nasopharyngeal carcinoma cells. The authors showed that the synergism observed was independent of HDAC6 but rather through the induction of ROS-dependent estrogen receptor (ER) stress (100). The Class I HDACi, MGCD0103, has also been shown to synergize with proteasome inhibitors and induce apoptosis in Hodgkin lymphoma cell lines by an HDAC6-independent mechanism. The authors demonstrated that MGCD0103 resulted in the upregulation of several inflammatory cytokines, leading to the activation of nuclear factor (NF)-κB (NFκB) and attenuating tumor cell death. The subsequent inhibition of NFκB with proteasome inhibitors enhanced the MGCD0103-induced death of Hodgkin lymphoma cells (101). The combination of HDACi with proteasome inhibitors may additionally be used to treat glioblastomas in order to maximize the therapeutic efficacy and limit the toxicity associated with using proteasome inhibitors as a single agent in this disease (102). Clinical trials with bortezomib in combination with either vorinostat as a third-line treatment in advanced NSCLC (ClinicalTrial.gov identifier: NCT00798720) or with panobinostat for the treatment of relapsed/refractory T-cell lymphoma or NK/T-cell lymphoma following conventional chemotherapy failure (ClinicalTrial.gov identifier: NCT00901147) have been completed.

Carfilzomib is a second-generation proteasome inhibitor that was recently approved by the FDA for the treatment of relapsed and refractory multiple myeloma, in patients who were given at least two prior therapies (103, 104). There is an ongoing phase I/II clinical trial with panobinostat and carfilzomib in patients with relapsed/refractory multiple myeloma (ClinicalTrial.gov identifier: NCT01496118) and a completed phase I clinical trial of carfilzomib with vorinostat for the treatment of relapsed/refractory B-cell lymphomas (ClinicalTrial.gov identifier: NCT01276717).

## Combining HDACi with Hormonal Therapy

Dysregulation of hormone signaling is seen in many cancers and is an essential component of carcinogenesis. Strategies inhibiting estrogen and androgen signaling in breast and prostate cancers, respectively, have shown clinical success (105, 106). Aberrant acetylation and HDAC expression has been observed in both breast and prostate cancer cell lines and patient tumors (107, 108). Thus, the combination of HDACi and hormonal therapy is under investigation in both clinical and preclinical settings for the treatment of breast and prostate cancers. While vorinostat as a monotherapy was not found to be effective against the treatment of MBC (109), in combination therapy with the anti-estrogen, tamoxifen, it has shown great promise (57). Hormone therapy resistance is a challenging issue for treating ER positive breast cancers and thus, combination therapy with HDACi has been studied. Researchers carried out the first clinical trial combining vorinostat and tamoxifen for hormone therapy-resistant breast cancer (ClinicalTrial.gov identifier: NCT00365599) (67). This study was for advanced breast cancer patients who progressed on prior hormone therapy, as an approach to restore hormone sensitivity to tamoxifen. Vorinostat-induced H4 acetylation and HDAC2 expression was performed in peripheral blood mononuclear cells on the 43 patients recruited in this study. Overall, this study demonstrated that the addition of the HDACi, vorinostat, to tamoxifen hormone receptor-positive breast cancers lead to tumor regression or prolonged disease stabilization in 40% of patients who had progressed on prior hormonal therapy and chemotherapy. Additionally, this study confirms that HDAC2 expression is a predictive marker for response and that histone hyperacetylation may be an effective pharmocodynamic marker for determining the efficacy of vorinostat and tamoxifen combination therapy.

While HDACi as a monotherapy in prostate cancer has not shown promise in clinical trials (110–112), in combination with the antiandrogen, bicalutamide, a synergistic cytotoxicity was observed in preclinical models (113–115). There have been two completed trials evaluating the combination of HDACi and hormonal therapy in prostate cancer (116). The initial trial was a phase II trial with vorinostat in combination with bicalutamide and radical prostatectomy, in patients with localized prostate cancer (ClinicalTrial.gov identifier: NCT00589472) (117). The second clinical trial was a phase I/II clinical trial studying the safety and efficacy of panobinostat and bicalutamide in patients with recurrent prostate cancer after castration (ClinicalTrial.gov identifier: NCT00878436). While the combination of HDACi with androgen-deprivation therapy holds much promise, the advancement to phase III clinical trials will define the fate of HDACi in the treatment of prostate cancer.

## Combination of HDACi with Tyrosine Kinase Pathway Inhibitors

Receptor tyrosine kinase (RTK) activation regulates a vast range of biological functions, such as cell growth, survival, organ morphogenesis, neovascularization, and tissue regeneration and repair. While the activity of RTK is regulated in normal cells, RTK signaling is dysregulated in many cancers. Therefore, RTKs are promising therapeutic targets (118, 119). Activation of RTK has been demonstrated to activate RAS-RAF-MEK-MAPK and PI3K-AKT pathways among others. This results in an increased expression of c-Myc and cyclin D1 oncogenes and lowered activation of proteins within the cell cycle checkpoint leading to enhanced cell cycle progression and survival. Monoclonal antibodies or RTK inhibitors of these pathways may block cell cycle progression, inhibit production of pro-angiogenic factors and induce apoptosis in various *in vitro* and xenograft models (120). Interestingly, cyclin D1 interacts with class I/II HDACs and HDACs regulate the expression of c-Myc and cyclin D1. Additionally, cell treatments with HDACi reduced cyclin D1 transcription and increased c-Myc degradation. Therefore, the combination of RTK targeted therapies and HDACi represents a novel approach for targeted cancer therapy (57, 121). Sorafenib (BAY 43-9006) is a novel multikinase inhibitor which has been shown to block the RAS-RAF-MEK-MAPK pathway. Recent work has demonstrated that vorinostat and sorafenib synergistically kill tumor cells (122). Numerous phase I clinical trials have been conducted with the combination of sorafenib and HDACi for treating patients with advanced/metastatic solid malignancies and refractory/relapsed acute myeloid leukemia (AML), soft tissue sarcomas, and lung, advanced liver, and renal cancers (ClinicalTrial.gov identifier: NCT01159301, NCT00823290, NCT01005797, NCT01075113, NCT00635791). Gefitinib (Iressa) is a selective, first generation, reversible EGFR tyrosine kinase inhibitor (123). Studies have demonstrated that gefitinib combined with HDACi synergistically induces growth inhibition and apoptosis in gefitinib-resistant NSCLC cells (124). Currently, there is a phase I/II clinical trial combining Gefitinib and vorinostat in relapsed/refractory patients with advanced NSCLC (ClinicalTrial.gov identifier: NCT01027676) and phase I study of vorinostat combined with gefitinib in patients with BIM polymorphysim associated resistant EGFR mutant lung cancer (ClinicalTrial.gov identifier: NCT02151721). Additionally, the TRK inhibitor, trastuzumab, a monoclonal antibody, shown to target HER2 and downregulate the PI3K-AKT pathway, was evaluated in combination with panobinostat in a phase I/II clinical trial in adult female patients with HER2 positive MBC whose disease has progressed on or after trastuzumab. Unfortunately, this trial was prematurely terminated as a result of poor efficacy (ClinicalTrial.gov identifier: NCT00567879). The TRK inhibitor, everolimus, which is an mTOR serine/threonine protein kinase inhibitor, is currently being evaluated in the clinic with panobinostat for the treatment of recurrent multiple myeloma, non-Hodgkin and Hodgkin lymphoma (ClinicalTrial.gov identifier: NCT00918333) and in solid tumors/lymphomas with enrichment for EBV-driven tumors (ClinicalTrial.gov identifier: NCT01341834).

## HDACi in Combination with Other Epigenetic Modifiers

Aberrant DNA methylation has emerged as a distinct molecular pathway leading to malignant transformation. The hypomethylating agents, azacitidine and decitabine, are active anticancer drugs currently FDA approved for treating AML, chronic myelomonocytic leukemia, and myelodysplatic syndromes (MDSs) (125, 126). The dual inhibition of HDACi with DNA-hypomethylating agents is a growing area of interest in the clinic. Recent *in vitro* work has demonstrated that the combination of valproic acid and decitabine synergistically inhibits growth and induces apoptosis in the leukemia cells lines, HL-60, and MOLT4 (127). A subsequent phase I/II clinical trial with valproic acid and decitabine included 54 patients with leukemia. In the study, 12 patients showed objective responses including 10 complete remissions and a large cytogenetic response was seen in 6 of the 8 responding patients. Hypomethylation of *p15*, a central cell cycle regulating gene, proved to be the best indicator of response. This study demonstrated that the combination was safe and resulted in a transient reversal of aberrant epigenetic markers (128) (ClinicalTrial.gov identifier: NCT00075010). Two phase I clinical trials were carried out to assess the pharmacokinetics of 5-azacitidine administered with the HDACi, phenylbutyrate, for the treatment of refractory solid tumors or hematological malignancies (129). 5-azacitidine was administered once daily as a subcutaneous injection, while varying doses of phenylbutyrate were administered as a continuous intravenous infusion. This study showed that 5-azacitidine was rapidly absorbed and eliminated *via* subcutaneous injection and sufficient 5-azacitidine exposure was seen leading to the pharmacodynamic effects in tumors. Additionally, a phase I clinical study was carried out in MDS or AML patients receiving 5-azacitidine followed by sodium phenylbutyrate. In the trial, 11 of the 29 patients responded; 6 among the 6 responding patients with pretreatment methylation of *p15* or *CDH-1* promoters saw a reversal of methylation during the first cycle of therapy, while zero among the six non-responding patients studied showed any demethylation. This study showed that the molecular mechanism responsible for responses to DNA methyltransferase and HDACi combination therapy might include the reversal of aberrant epigenetic gene silencing (130) (ClinicalTrial.gov identifier: NCT00004871).

## Combining HDACi with Immune Checkpoint Inhibitors

Immune checkpoint inhibitors have transformed the way in which solid tumors and hematological malignancies are treated. The immune checkpoint proteins, cytotoxic T lymphocyte-associated antigen 4 (CTLA-4) and programmed cell death receptor 1 (PD-1), have been shown to be negative regulators of T-cell immune function. PD-1 regulates T-cell activation through binding to its ligands, PD-L1 and PD-L2. Inhibiting the PD-1 and PD-L1/L2 interaction results in enhanced T cell activation and effector functions, leading to an increased activation of the immune response. At present, there are four immune checkpoint inhibitors approved by the FDA, ipilimumab (an anti-CTLA-4 drug), nivolumab and pembrolizumab (anti-PD-1 drugs), and the anti-PDL1 agents, such as atezolizumab, durvalumab, and avelumab (131, 132). There are over 500 ongoing clinical trials using immune checkpoint inhibitors for the treatment of various cancers including resected, localized, and advanced solid tumors and hematological malignancies. Despite promising results with immune checkpoint blockade therapy, solid and hematological malignancies often evade the host immune system and combination therapy with other chemotherapeutic drugs is an avenue to overcome these limitations. HDACi have been shown to have potent immunomodulatory activity, rationalizing their use in cancer immunotherapies. The combination of immune checkpoint inhibitors with HDACi has shown promising results both *in vitro* and *in vivo* (133, 134). HDACi enhance the immunotherapy response (135) and augment immunotherapy with PD-1 blockade in melanoma cells (136), inhibit apoptosis of CD4^+^ T cells within the tumor, upregulate antitumor immune responses and restrict tumor growth (137). It was recently shown in mice that cancers resistant to the immune checkpoint inhibitors, anti-PD-1 and anti-CTLA-4, could be cured by eliminating myeloid-derived suppressor cells (MDSCs) (138). The authors demonstrated that the HDACi, entinostat, targeted the mouse granulocytic MDSCs, responsible for the resistance to immune checkpoint blockade seen. In a mouse model of lung and renal cell carcinoma, entinostat was additionally shown to improve the antitumor effect of PD-1 targeting by inhibiting MDSC function (139). Furthermore, in breast cancer patients, entinostat reduced MDSCs and the modulation of MDSC CD40 expression and additionally, increased HLA-DR expression on CD14^+^ monocytes in the cancer patients. These results establish a rationale for combination therapy between entinostat and immune checkpoint inhibitors (140). It was recently shown that the prostate cell line, LNCAP, and the breast cell line, MDA-MB-231, were more sensitive to T-cell mediated lysis following treatment with the HDACi, entinostat, or vorinostat. The authors demonstrated that HDAC1 played a central in the reversal of carcinoma immune escape through the induction of ER stress, resulting in the activation of the unfolded response, subsequently leading to immunogenic modulation and increased tumor sensitivity to T-cell mediated lysis (141).

At present, there are several ongoing clinical trials combing HDACi with immunotherapy strategies. They include the immune checkpoint inhibitor, pembrolizumab, which is currently being evaluated in the clinic in combination with vorinostat for the treatment of advanced renal or urothelial cell carcinoma (ClinicalTrial.gov identifier: NCT02619253). Likewise, pembrolizumab is being evaluated with entinostat for treating metastatic melanoma of the eye (ClinicalTrial.gov identifier: NCT02697630), while the monoclonal anti-PD-1 antibody, nivolumab, is being studied in combination with azacitidine and entinostat in metastatic NSCLC patients (ClinicalTrial.gov identifier: NCT01928576). A phase I/II study assessing entinostat in combination with interleukin 2 for the treatment of metastatic renal carcinoma was recently conducted with promising clinical activity (ClinicalTrial.gov identifier: NCT01038778). Of the 47 patients who took part in the study, an objective response of 37% was observed, a 13.8-month median progression-free survival as seen and 65.3 months was the overall survival (142). Additionally, a phase III trial evaluating endocrine therapy with the HDACi, entinostat, or placebo in hormone receptor-positive breast cancer patients is about to commence (ClinicalTrial.gov identifier: NCT02115282) (140).

A summary of HDACi in combination therapy with anticancer agents in ongoing or completed clinical trials, for the treatment of cancer is discussed in Table 3.

**Table 3:** Histone deacetylase inhibitors (HDACi) in combination with other anticancer agents: phase I/II/III clinical trials.

HDACi	Combination(s)	Cancer(s)	Phase I/II/III clinical trial
Vorinostat (SAHA)	Olaprarib, gemcitabine, busulfan and melphalan	Hodgkin’s or non-Hodgkin’s lymphoma	I/II
	Radiotherapy	Gastrointestinal cancer	I
	Radiotherapy	Non-small cell lung (NSCLC)	I
	Radiotherapy and capeceitabine	Non-metastatic pancreatic	I
	Doxorubicin	Advanced solid tumors	I
	Pegylated liposomal doxorubicin	Relapsed or refractory lymphoma	I/II
	Doxorubicin hydrochloride liposome and bortezomib	Relapsed or refractory multiple myeloma	I
	Carboplatin and paclitaxel	Advanced solid malignancies	I
	Carboplatin, paclitaxel, placebo	NSCLC	II
	Carboplatin or paclitaxel	Advanced solid tumors	I
	Bortezomib	NSCLC	II
	Carfilzomib	Relapsed/refractort B-cell lymphoma	I
	Tamoxifen	Breast	II
	Tamoxifen and pembrolizumab	Breast	II
	Bacalutamide and radical prostatectomy	Prostate	II
	Gefitinib	Relapsed or refractory NSCLC	I/II
	Sorafenib	Advanced liver	I
	Pembrolizumab	Renal or urothelial cell carcinoma	I
	Pembrolizumab	NSCLC	I/II
	Pembrolizumab	Breast	II
	Rituximab	Lymphoma/leukemia	II

Valproic acid	Temozolomide and radiation	Brain	II
	Epirubicin	Advanced solid tumors	I
	Epirubicin/FEC	Breast	I/II
	Decitabine	Leukemia	I/II

Panobinostat (LBH589)	Radiotherapy	Prostate, esophageal, and head and neck	I
	Carfilzomib	Relapsed/refractory multiple myeloma	I/II
	Bortezomib	Relapsed/refractory T-cell lymphoma (TCL) or NK/TCL	II
	Bicalutamide	Prostate	I/II
	Sorafenib	Hepatocellular carcinoma, kidney and soft tissue carcinoma	I
	Everolimus	Multiple myeloma, non-Hodgkin or Hodgkin lymphoma	I/II
	Ipilimumab	Melanoma	I

Romidepsin (FK288)	Carfilzomib	Relapsed/refractory PTCL	I/II
	Gemcitabine, dexamethasone and cisplatin	PTCL and diffuse large B-cell lymphoma	I
	5-azacitidine	Relapsed/refractory lymphoid maligancies	I/II

Belinostat (PXD101)	Doxorubicin	Soft tissue sacrcoma	I/II
	Carboplatin and/or paclitaxel	Solid tumors	I

Entinostat (MS275-SNDX-275)	Sorafenib tosylate	Solid tumors or acute myeloid leukemia (AML)	I
	Exemestane	Breast	I
	Pembrolizumab	Advanced solid tumors	I
	Pembrolizumab	Metastatic melanoma of the eye	II
	Nivolumab	NSCLC	II
	Avelumab	Epithelial ovarian	I/II
	Atezolizumab	Breast	I/II

Sodium phenylbutyrate (4-PBA)	Azacitidine	AML or myelodysplastic syndrome (MDS)	I

Tacedinaline (CI994)	Gemcitabine	Advanced NSCL	III
	Gemcitabine	Advanced pancreatic	II

Mocetinostat (MGCD0103)	Docetaxel	Advanced tumors	I
	Brentuximab vedotin (SGN-35)	Relapsed/refractory Hodgkin lymphoma	I/II
	Azaxitidine	High-risk MDS or acute myelogenous leukemia	I/II
	Durvalumab	Squamous cell carcinoma or the oral cavity	I

Abexinostat (PCI24781)	Doxorubicin	Soft tissue sarcoma	I/II
	Pazopanib	Metastatic solid tumors	I

Resminostat (4SC201)	Sorafenib	Hepatocellular carcinoma	I/II

Quisinostat (JNJ-26481585)	Paclitaxel and carboplatin	Ovarian	II

HBI-8000	Nivolumab	Melanoma, renal cell carcinoma, and NSCLC	I/II

CUDC-101	Cisplatin	Head and neck	I

AR42	Decitabine	AML	I
	Pomalidomide	Relapsed multiple myeloma	I

4SC202	Pembrolizumab	Malignant melanoma	I/II
CG200745	Gemcitabine and erlotinib	Advanced pancreatic	I/II
ME-344	Toptecan	Solid tumors	I/II

*NIH clinical trial database: www.clinicaltrials.gov*.

## Side Effects and the Search for Biomarkers of HDACi

Like other therapeutics, HDACi are associated with side effects. These include thrombocytopenia, neutropenia, nausea, vomiting, diarrhea, and fatigue. The most troubling effect is cardiac toxicity, including ventricular arrhythmia. Indeed, prior to FDA approval, there were six deaths in patients treated with romidepsin (143–145). In order to minimize the off-target toxicity of HDACi, tissue and cell targeted delivery of HDACi and the identification of isoform selective HDACi will become the focus of future research into HDACi.

In an ideal setting, clinical trials would use biomarkers to select patients or predict their responses to HDACi and to limit adverse effects in particular by excluding patients unlikely to benefit from therapy. Recent work has demonstrated that knockdown of HDAC1 and not HDAC2 or HDAC3 resulted in an increased resistance to belinostat-induced cell death in HeLa cells (146). While this data is suggestive for high HDAC1 levels to correlate with sensitivity to HDACi treatment, more experimental data is required to determine whether a tumor-specific HDAC isoenzyme profile may predict the response to individual HDACi. Further research has shown that the molecular profiling of NSCLC cells may be of value in selecting patients for HDACi therapy. The study identified a nine-gene RNA expression signature useful in predicting trichostatin A or vorinostat-induced apoptosis and may lead to individualized treatment for patients with NSCLC (147). Additionally, further studies showed increased nuclear STAT1 and phospho-STAT3 staining in CTCL cells was associated with a lack of clinical response to vorinostat. The authors concluded that blocking this pathway may lead to improvements in the response to vorinostat and may indeed be predictive of response of CTCL patients to the agent (148).

## Conclusion and Future Directions

The HDACi, vorinostat, and romidepsin are FDA approved for the treatment of CTCL, while belinostat and panobinostat are approved for the treatment of PTCL and multiple myeloma, respectively. At present, the most prominent treatment option for cancers is therapy that induces DNA damage. However, lack of response and development of resistance to the treatment is an issue. The approval of vorinostat, romidepsin, belinostat, and panobinostat has promoted HDACi as a routine therapeutic approach for the treatment of an ever-increasing list of cancers. Using HDACi as chemosensitizers that increase the efficiency of other chemotherapeutic compounds has shown great promise in preclinical and clinical trials. The combination of drugs targeting DNA repair pathways and HDACi holds great promise. While there are numerous DNA repair pathway-targeting drugs currently in clinical trials (149), their combination with HDACi have yet to be tested. Radiotherapy, topoisomerase inhibitors, and platinum-based chemotherapeutics, all of which cause cell death through the induction of DNA damage, are currently being evaluated in clinical trials in combination with HDACi. In preclinical and clinical settings, HDACi have shown synergistic or additive antitumor effects with numerous chemotherapeutic agents. The combinations of HDACi with proteasome inhibitors, hormonal therapy, tyrosine kinase inhibitors, DNA-hypomethylating agents, and immune checkpoint inhibitors in clinical trials will define the fate of HDACi in the years to come. In recent years, immune checkpoint inhibitors have played a critical role in the treatment of solid tumors and hematological malignancies. Their combination with HDACi have shown significant synergy in both in preclinical and clinical settings and considered a major breakthrough in the treatment of cancer.

Over the last few decades, we have seen an expansion in our understanding of the epigenetic regulation of normal and cancer cells. As epigenetic changes that occur during the development of cancer are reversible and amenable to pharmacological intervention, HDACi provide a unique avenue of treating cancers (150). Over the last few years we have witnessed the approval of HDACi by the FDA, the approval of the PARP inhibitor, olaparib, the proteasome inhibitor, cafilzomib, and in 2011 the approval of the first immune checkpoint inhibitor, ipilimumab. While HDACi alone and in combination with other anticancer agents have revolutionized the way cancers are being treated, they have been met with limitations. Cancer cell resistance to HDACi and the toxic effects associated with HDACi need to be overcome and larger, multicentric clinical trials are necessary to fully elucidate the benefits of HDACi combination therapy. Future studies will entail improving the selectivity of HDACi to amplify their accumulation in cancer cells at a lower dose and thereby reduce the toxic effect of these drugs on normal healthy cells. Furthermore, the identification of biomarkers for HDACi, alone and in combination with other anticancer agents is imperative in order to predict the response of the individual patient to treatment. In the years to come, genome sequencing may enable the delivery of personalized care to patients by either selecting or predicting an individual patient’s response to the cancer treatment.

## Author Contributions

All authors were involved in the conception of the manuscript, the drafting and/or critically reviewing of the manuscript and have approved the final version for publication.

## Conflict of Interest Statement

The authors declare that the research was conducted in the absence of any commercial or financial relationships that could be construed as a potential conflict of interest.
